# Pesticide Residues Analysis in Iranian Fruits and Vegetables by Gas Chromatography-Mass Spectrometry

**Published:** 2019

**Authors:** Zahra Hadian, Samira Eslamizad, Hassan Yazdanpanah

**Affiliations:** a *Department of Food Science and Technology, National Nutrition and Food Technology Research Institute, Shahid Beheshti University of Medical Sciences, Tehran, Iran.*; b *Food Safety Research Center, Shahid Beheshti University of Medical Sciences, Tehran, IR Iran. *; c *Department of Toxicology and Pharmacology, School of Pharmacy, Shahid Beheshti University of Medical Sciences, Tehran, IR Iran.*

**Keywords:** Gas Chromatography-Mass Spectrometry, Pesticide residues, Fruits, Vegetables, Iran

## Abstract

Pesticide residues in fruits and vegetables are one of the highest concerns of consumers who need food safety. In this study, forty-eight pesticide residues from different chemical structures including organochlorine, organophosphorus, organonitrogen, dicarboximides, strobilurin, triazine, pyrethroids, and other chemical groups. In 85 fruits and vegetables were determined and confirmed by GC-MS. The pesticide was extracted with ethyl-acetate, then, the extracts cleaned using high performance gel permeation column chromatography (GPC) and solid phase column (SPE). The mean recoveries of the pesticides were between 81 and 136%. The reproducibility of the relative standard deviation values was 2.1% and 14.8%. Pesticide residues were more frequently found in vegetables (65.5%) than in fruits (26.7%). The limits of detection and quantification of pesticide residues for the method were ranged from 0.003 to 0.06 μg/g and between 0.01 to 0.1 μg/g respectively. The analyzed samples did not contain residues from the monitored pesticides that were higher than the accepted maximum residue limits (MRLs) as adapted by the FAO/WHO Codex Alimentarius Commission.

## Introduction

The use of pesticides in agriculture has undoubtedly increased the crop yield and reduced crop losses (1-3). On a worldwide basis, pre-and post-harvest crop losses are as much as 45%. The use of pesticides in modern agriculture has seen increased yields and more predictable food production, reduction in labor, and lower acreages in production to yield a given quantity of food. This may give rise to ecological concerns when toxicity occurs in non-target organism; exposures exceed expected amounts, or drift damages off environments (3, 4). Many studies including estimates of human safety are required before a pesticide can be registered for use (2, 5). Pesticides are utilized on a wide variety of crops, and their residues are detected in with different methods. Over 4 billion pounds of pesticides are used on crops annually worldwide. In recent years, increasing public concern about possible health risks from pesticide residues in the diet has significantly improved the strategies for crop protection, affecting the food quality and safety. Additionally, there has been widespread concern for health in society, resulting in strict regulation of the maximum residue limits (MRLs) and total dietary intakes of pesticide residues in food commodities (6-8). The analysis of fruit and vegetables must be rapid and accurate, due to the complexity of the included matrices, extraction is usually accomplished by cleaning the samples before performing gas chromatographic analysis (9). A broad variety of solvent extraction and partitioning systems have been used for crop sample extractions; for example, acetone, methanol, acetonitrile, and ethyl acetate are commonly applied in solvent-based extraction methods (10, 11). Due to the broad range of physicochemical properties of the target pesticides (12), many procedures are based on the use of extensive cleanup of extract, such as liquid-liquid extraction (LLE) or supercritical extraction, solid phase extraction (SPE) and gel permeation chromatography (GPC).

Separation and cleanup procedure should supply a good isolation of the pesticide from the matrix, proceeding by the necessary cleaning of crude vegetable and fruit extracts. Optimum isolation and cleaning procedures provide the separation of pesticide residues from interfering components of the matrix that have adverse effect on the limits of quantitation (LOD) of the analytical method (13).

GC-MS has recently been generally accepted in the pesticide analysis field because it simultaneously allows for determination and confirmation of a large number of compounds. Additionally, it has low detection limits as a consequence of its high selectivity from the use of the selected ion monitoring. However, current observations have confirmed that GC-MS with an ion trap (IT) detector is suitable for rapid semi-quantitative screening. Pesticides have a wide variety of properties, such as solubility, volatility, and stability. Many sample matrix components are present in the GC-MS test solution to limit pesticide loss under the sample preparation conditions (13- 22). 

High levels of matrix components in GC-MS test solutions often suppress or promote ionization of the pesticide, interfering with the determination of pesticides in ion trap chromatograms. Furthermore, under analytical conditions for evaluating over 100 pesticides, some pesticide peaks might overlap each other in the ion trap chromatograms. Therefore, the analysis could lead to false positive and/or false negative results. Moreover, when many test solutions are analyzed sequentially by GC-MS, there could be a lack of resolution and sensitivity from a dirty injection port, separation column, or ion source. Thus, it is still a great challenge to develop a method for extracting the information of specific components from a multicomponent overlapping signal (10, 22).

The research has shown that the asymmetric nature (polar and nonpolar) of insecticides, fungicides, and herbicides involve to considering the selectivity of various cleaning methods. Co-extractive GPC and SPE cleanup method cause to distinguish high-efficiency and identify pesticide residues (23).

Gel-permeation chromatographic (GPC) cleanup is used for the extraction of lipids and epicuticular wax material, which must be eliminated before the determinative stage. There are more than 1000 pesticides used around the world to ensure food is not damaged or destroyed by pests. Each pesticide has different properties and toxicological effects. Many of the older, cheaper (off-patent) pesticides, such as dichlorodiphenyltrichloroethane (DDT) and lindane, can remain for years in soil and water. These chemicals have been banned by countries who signed the 2001 Stockholm Convention (12). 

**Table 1:** Validation parameters for determination of pesticide residues in fruit and vegetable samples by GC-MS method (n = 5)

**Pesticide**	**Classes OC/OP/O** *	**LOD** a **(μg/g-1)**	**LOQ** b **(μg/g)**		**Recovery (%)**
DDD-pp	OC	0.003	0.01		114
DDE-pp	OC	0.003	0.01		99
DDT-op	OC	0.003	0.05		136
DDT-pp	OC	0.003	0.05		122
HCH-γ	OC	0.003	0.01		95
atrazine	O	0.009	0.03		106
azinphos-methyl	OP	0.003	0.01		111
chlorpyrifos	OP	0.009	0.03		106
chlorpyrifos-methyl	OP	0.003	0.01		101
diazinon	OP	0.003	0.01		101
dichlorvos	OP	0.003	0.01		90
dicofol	OC	0.015	0.05		115
endosulfan (I)	OC	0.003	0.01		106
endosulfan (II)	OC	0.003	0.01		103
endosulfan-sulphate	OC	0.003	0.01		108
ethion	OP	0.012	0.04		112
ethoprophos	OP	0.015	0.05		88
etrimfos	OP	0.003	0.01		96
fenitrothion	OP	0.003	0.01		95
fenpropathrin	O	0.003	0.01		104
fenvalerate	O	0.003	0.01		109
fonofos	OP	0.003	0.01		112
heptenophos	OP	0.009	0.03		91
hexachlorobenzene	OC	0.003	0.01		96
iprodione	O	0.009	0.03		113
isofenphos	OP	0.03	0.1		86
malathion	OP	0.06	0.02		98
mephosfolan	OP	0.003	0.01		106
methacrifos	OP	0.003	0.01		88
methidathion	OP	0.003	0.01		101
metribuzin	O	0.003	0.01		109
napropamide	O	0.003	0.01		88
parathion-methyl	OP	0.012	0.04		86
pendimethalin	O	0.009	0.03		95
permethrin	O	0.003	0.01		97
phenthoate	OP	0.003	0.01		90
phosalone	OP	0.003	0.01		109
phosmet	OP	0.003	0.01		89
pirimiphos-ethyl	OP	0.009		0.03	104
pirimiphos-methyl	OP	0.003		0.01	103
profenofos	OP	0.003		0.01	115
prothiofos	OP	0.003		0.01	100
quinalphos	OP	0.003		0.01	114
simazine	O	0.012		0.04	105
tetrachlorvinphos	OP	0.015		0.05	106
triazophos	OP	0.003		0.01	110
trifluralin	O	0.003		0.01	81

* . Other pesticides

a . Limit of detection

b . Limit of quantification.

**Table 2:** Pesticide concentration in Iranian fruit and vegetable samples analysis by GC-MS method

**Sample**	**No. of samples**	**Pesticide**	**No. of positive samples**	**Mean ** **± ** **SD (mg/kg)**
Cucumber	15	Endosulfan I	5	0.03 ± 0.005
		Endosulfan II	5	0.03 ± 0.005
		Endosulfan-sulphate	5	0.04 ± 0.003
		Chlorpyrifos	5	0.03 ± 0.004
		Iprodione	5	0.02 ± 0.001
Carrot	5	Trifluralin	5	0.08 ± 0.006
Maskmelon	10	Endosulfan II	3	0.04 ± 0.003
		Endosulfan-sulphate	3	0.02 ± 0.001
Melon	10	Endosulfan-sulphate	2	0.06 ± 0.001
Tomato	15	Phosalone	5	0.05 ± 0.008
		Fenvalerate	1	0.05 ± 0.001
		Chlorpyrifos	10	0.03 ± 0.009
		Fenpropathrin	10	0.04 ± 0.009
		Permethrin	10	0.14 ± 0.017
		Fenvalerate	10	0.03 ± 0.005
		Iprodione	10	0.03 ± 0.006
Watermelons	10	ND^b^	0	
Cabbage	5	ND	0	
Lettuce	5	ND	0	

a . SD = Standard deviation;

b . ND. Pesticide residue not detected.

**Table 3:** Average recoveries (%) and relative standard deviations (%) of pesticide in Iranian fruits and vegetables with GC-MS method (n = 5)

**Cucumber**		**Melon**		**Tomato**		**Maskmelon**		**Carrot**		**Sample**
**RSD** **(%)**	**Average recovery (%)**	**RSD** **(%)**	**Average** **recovery (%)**	**RSD** **(%)**	**Average recovery (%)**	**RSD** **(%)**	**Average recovery (%)**	**RSD** ^a^ **(%)**	**Average recovery** **(%)**	**Pesticide**
7.7	96.3	8.8	99.5	11.5	85.3	7.7	119.5	8.8	89.5	Chloropyrifos
12.0	108.3	6.5	101.0	5.5	87.0	14.0	105.1	6.5	112.0	Endosulfan-I
4.4	104.0	5.4	85.5	11.9	84.0	4.4	119.7	5.4	108.5	Endosulfan-II
14.5	93.4	13.5	121.4	21.5	117.0	14.8	107.1	10.5	91.0	Endosulfan-sulphate
6.3	97.3	4.9	79.0	7.2	89.7	6.01	84.8	4.5	106.2	Fenpropathrin
14.6	84.0	7.7	104.2	3.5	95.7	14.5	101.5	7.3	104.4	Fenvalerate
6. 1	100.3	6.1	110.5	4.0	112.8	6.01	106.5	7.1	85.5	Iprodione
8.8	85.0	2.1	93.5	2.5	102.3	8.9	79.5	6.3	98.6	Permethrin
6.2	127.6	13.5	103.3	4. 2	94.33	6.2	83.5	11.5	118.5	Phosalone
11.3	108.0	10.6	88.0	6.7	100.7	11.5	87.0	8.5	98.0	Trifluralin

a . Relative standard deviation

Fruit trees were cultivated in Iran on about 2.2 million hectares with about 17 million tonnes of yield in 2014. Approximately 80% of pesticides application for crop protection in Iran is from June to the end of September. Control situation of agriculture crops are 2 types and for chemical control and non-chemical control these values are 10,203,207 hectares (83%) and 2,127,530 hectares (17%), respectively. The area under the vegetable and its cultivation in Iran is about 1 million hectares, 600 hectares in the garden area and about 10 thousand hectares of which are also greenhouses and in Tehran province the cultivated area of vegetables accounts one thousand and 787 hectares with the production of 504 thousand and 881 tons. Approximately, 80% of pesticides application for crop protection in Iran is from June to the end of September. In a research conducted by Morteza *et al.* (2017), the average annual volume of the active ingredients of all pesticides used during the period 2012-2014 was calculated. This study showed that on average about 14,000 tonnes of agriculture pesticides, expressed in active ingredients (AI), were annually used in Iran. Herbicides constituted the largest volume (43%), followed by insecticides, acaricides (37%), and fungicides (19%) (24).

The benefits of national monitoring programs are enhanced food safety, awareness of contamination problems, and availability of intake data for assessing health hazards, improved management, and use of natural resources and understanding of a series of measures for good agricultural practice. Due to the use of pesticides for protection of crops the contamination of pesticide residues could be recognized as an important public health problem and in order to promise food safety for consumers, the present study was undertaken to determine different synthetic agrochemicals such as insecticides, fungicides, and herbicides residues in fruit and vegetables collected from the main market in Tehran. Additionally, depending on the nature of pesticide residues, samples cleanup processing was performed by GPC and SPE procedures and followed by quantitative analyses with GC-MS. 

## Experimental

*Chemicals and reagents*

All organic solvents were obtained from Fisher (Fair Lawn, NJ UK). Sodium chloride (≥ 99%), anhydrous sodium sulfate (≥ 99%), dipotassium hydrogen phosphate (≥ 98%), sodium bicarbonate in powder form and ACS grade (≥ 98%) were obtained from Aldrich (Milwaukee, WI. US). Purified water was prepared from Milli-Q water purification system (Millipore, Beford, MA). The purity of pesticide standards and internal standard solutions were ≥ 95% and were obtained from Restek (Thames Restek CO., UK). 

Phosphate buffer (1 mol/L) was prepared by dissolving 105 g of dipotassium hydrogen phosphate and 61 g of potassium dihydrogen phosphate in 1 L of water.

Stock standard solutions of 47 organochlorine pesticides (OC), organophosphorus pesticides (OP) and other pesticides were prepared in ethyl acetate at concentrations ranging from 50 to 200 ng/μL. Stock pesticide solutions were kept at 4 °C in a dark place until analysis. Spiking solutions contain pesticides at concentrations of 10–50 ng/μL. Aldrin solution at 50 ng/μL in acetone was used as internal standard. 

*Sampling*

A total of 85 samples including 10 watermelons (*Citrullus lunatus*), 10 muskmelons (*Cucumis melo L.*), 10 melons (*Cucumis melo*), 20 cucumbers (*Cucumis sativum*), 5 carrots (*Daucus carota*), 5 lettuces (*L.S. Var. Longifolia*), 20 tomatos (*Solanum lycopersicum*), and 5 cabbages (*Brassica oleraceae var. capitate* *f. alba*) were obtained from wholesale fruit and vegetable center in Tehran. Sampling and transporting of fresh fruit and vegetables were performed in accordance with the general principles and methods of the Codex Alimentarius 2000 (25). 47 pesticides were subjected to analysis, including organochlorine, organophosphorus, organonitrogen, dicarboximides, strobilurin, triazine, pyrethroids, and other chemical groups. Two kilograms of each sample were collected and prepared for residue analysis according to the European Commission (EC) directive 91/414 method (26, 27). All samples were packaged in plastic bag and cooled by storing at -18 °C before analysis.

*Sample preparation*

For analysis, 30 g of chopped sample was blended and dissolved in 60 mL of ethyl acetate then mixed with 5 g of sodium bicarbonate and 40 g of anhydrous sodium sulfate (28). The subsample was mixed for 2 min using an Ultra Turrax homogenizer (IKA T10 Cole-Parmer, US) and followed by centrifugation at 3000 rpm for 5 min at -10 °C (Becton, Dickinson, & Co., Parsippany, NJ, US). The extract was evaporated to 0.5 mL under nitrogen gas. Co-extractives are performed by passing sample through SPE and GPC columns. Envirosep-ABC (Phenomenex J2, Scientific, Colombia, USA) 60×21.2 mm i.d. columns were used in series. A 2-mL aliquot of crude-extract (i.e., equivalent of l g of original matrix) was loaded onto the HPGPC.

The flow-rate of the mobile phase (cyclohexan:ethyl acetate, 1:1, v v^-1^) was 5 mL min^-1^. The dump time was 16 min; then, the collected sample (14 mL) was evaporated to dryness using a rotary evaporator. The remaining solvent was dried using a gentle stream of nitrogen and the remainder was dissolved in l mL of toluene prior to GC analysis. The pesticide fraction obtained by HPGPC cleaning of the crude extract was evaporated to dryness using a mild nitrogen stream.

The residue was then dissolved in 10 mL ethyl acetate. To prepare “spiked sample”, a 5-mL aliquot, was carefully evaporated and the reminder was dissolved in 0.5 mL of toluene. The SPE cartridge used in the study was 500 mg, and it also consisted of 6 mL of ENVI-Carb graphitized carbon black (GCB) sorbent (Supelco, Bellefonte, PA, USA).

The cleanup procedure was conducted in 5 replicates and there was a control in which no cleanup was performed; these were set aside for later analysis. Ethyl acetate extract (10 mL) was passed through a preconditioned cartridge and the extract was collected. After filtration to remove the precipitated proteins and solids, the pH of the sample was adjusted.

 The collected eluate was evaporated to <1 mL with the aid of a nitrogen stream at 40 °C. Then, acetone (10 mL) was added, and the eluate was again evaporated under nitrogen to <1.0 mL. Add 50 μL internal standard (1.0 ng/μL, final concentration of aldrin), and dilute extract to 2.5 mL with acetone and 20 μL spiking solution (10 ng/μL for pesticide mix solution) to 500 μL control extract (5 g sample per 2 mL ethyl acetate) to produce a final concentration of 1.0 ng/μL for Aldrin and 0.4 ng/μL for most compounds.

Finally for analysis 0.5 µL of the sample was injected into GC-MS. Limits of detection (LODs) of the method were reported by spiking samples at 0.1−1.0 mg/kg.

*Preparation of Standard Solutions*

Each stock solution of the pesticide standards was prepared at four concentration levels in ethyl acetate. Calibration standards were prepared by appropriate dilution of the stock solution in ethyl acetate at different levels (0.01- 0.5 mg/kg) in accordance with the European Commission guidelines (29).

The 47 mix pesticide standards were prepared in aceton (10 ppm). 8 μg/mL spiking solution was created by diluting 5 μL of the stock solution in 200 μL of acetonitrile (0.1% acetic acid). 200 μL of pesticide spiking solution was gently pipetted over each produce sample to create a final pesticide concentration of 100 ppb and the tubes were mixed for one minute to disperse the pesticides. The spiked samples were kept at refrigerator. 

*GC-MS Ion-trap*

GC-MS analysis of the fruit and vegetable samples for pesticide residues was carried out by using a Varian Model 3800 gas chromatograph (GC) fitted with an ion trap mass spectrometric ITMS Varian 2200 (Varian Instrument, CA, USA).

A DB-5 (Folsom, CA, USA) capillary column (25 m×0.25 mm I.D., 0.25-mm film thickness) was employed. The injections were performed with the column oven set at 100 °C in the splitless mode. That temperature was maintained for 1 min and then programmed at 10 °C min^-1^ to 200 °C and at 4 °C min^-1^ to 300 °C, where it was held for 3 min. The carried gas in the system was helium, which was delivered at flow rate of 1.0 mL/min.

The gas pressure program was set to 70 KPa (l min hold), 110 KPa at 8 KPa min^-1^, 150 KPa at 1 KPa min^-1^, and 174 KPa at 4 KPa min^-1^ (5 min hold). The Varian Saturn 2200 GC-MS and CP-3800 gas chromatograph were used. For ITMS, the temperature of the injector was 250 °C. The quantification matched calibrants were run with each batch at 5 levels.

*Statistical analysis*

Statistical analyses were performed using SPSS V.16 software for Windows (SPSS, Chicago, USA). One-way analysis of variance (ANOVA) procedure was used for statistical analysis. The results were reported significant at the 5% level (*P *< 0.05).

## Results and Discussion

The GC-MS parameters for each pesticide are illustrated in Table 1. A total of 85 samples from different types of fruit and vegetables were examined for 47 pesticide residues using GC-MS. HPGPC and SPE were used in our experiments as co-cleanup techniques that provided good separation of bulk plant co-extracts (pigments and cuticular waxes) from matrixes. The precision was estimated in terms of relative standard deviation (RSD) and ranged from 2.1% to14.8%.

The limits of detection and quantification of pesticide residues for the method were ranged from 0.003 to 0.06 μg/g and between 0.01 to 0.1 μg/g respectively. These results showed that this method was suitable for determination of the pesticide residues in fruit and vegetable samples. As **Figure 1** indicates 51.8% of the samples had no detectable pesticide residues. Of the analyzed samples, 48.2% contained detectable residues. No pesticides were detected in the cabbage, watermelon and lettuce samples.

The details of the detected residues and the mean and contamination ranges of the samples are given in Table 2. Pesticide residues were found in approximately 26.7% of the fruit samples, whereas 65.5% of the vegetable samples contained residues. Eleven pesticide residues were commonly detected in the analyzed samples. MRLs were not available from either the Codex or European Union. Ten of the 47 pesticide residues, including chlorpyrifos, endosulfan-II, endosulfan-I, endosulfan-sulfate, fenpropathrin, fenvalerate, iprodione, permethrin, phosalone, and trifluralin were detected in the analyzed samples.

The recovery of each pesticide residue is summarized in Table 3. In this research, the minimum LOD of the pesticides was defined as 0.003 μg/g that were estimated based on the noise levels on the chromatograms of the blank sample and the standard peaks. The results of the current study showed that no residues of the restricted or banned pesticides, such as DDT, HCH-γ and their metabolites were present in any of the analyzed samples. The residue levels from the monitored pesticides were significantly lower than the accepted maximum residue limits (MRLs) as adapted by the FAO/WHO (*P *< 0.001).

Yang *et al*. (2016) in a survey indicated that despite a high occurrence of pesticides in star fruit, wax apple, and Indian jujube (66.7%), the contamination levels do not contribute significantly to pesticide intakes and are unlikely to have public health effects (30). 

The results of the present study showed the pesticide residues were lower than the finding from Accra, Ghana that reported by Blankson *et al*. (2016) where 52% of the samples had detectable pesticide residues (21).

In a report from Ramezani *et al*., (2017) in Iran, the main pesticides residues detected in 2205 samples including cucurbits, some fruits and vegetable products in two seasons were as follows: Penconazole in 50 samples, Diazinon in 25 samples, Propargite in 19 samples, Iprodion in 16 samples, Fenvalerate in 5 samples, Oxydimeton methyl, D.D.T and Fenitrothion in 4 samples, Metalaxyl and Dichlorvos in 3 samples, Profenofos and Amitraz in 2 and Carbaryl, Ethion, Malathion and Tetradifon in 1 sample. The mean contamination of all the fruit types with residue levels higher than the MRLs was 2.1% (24).

Nasiri *et al*. (2016) in a research illustrated that among the 60 analyzed Iranian cucumbers samples, 41.7% of them were contaminated with pesticide residues which 31.7% of the samples had pesticide residues lower than maximum residue limit and 10% of samples had residue higher than maximum residue limit (11).

Okihashi *et al*. (2005) reported the similar result for determination of 180 pesticide residues in foods by GC-MS with the flame photometric detector. The average recoveries were good (above 80%) in all cases. The relative standard deviation values of the pesticides were < 5%. In comparison, in a monitoring program by the U.S. Food and Drug Administration, the rates of contamination in 1707 fruit and vegetable samples were 1.2% and 2.4%, respectively (31). The contamination rates of 149 pesticides in fruits and vegetables in the annual report of pesticide residues committee of FAO were 2% above the MRLs and 38% below the MRLs (32). The rates of violation and contamination reported for monitoring pesticide residues in Egyptian fruits and vegetables in 1997 demonstrated that the contamination percentages of the fruit and vegetable samples were 29.0% and 14.3%, respectively. Also, the violation rates in fruit and vegetables were 2.3% and 1.9%, respectively (33). 

Poulsen and Andersen (2003) monitored the pesticide residues in fruit and vegetables on the Danish market during 2000-01 and found that contamination rates above the MRLs in fruit and vegetables were 6% and 2%, respectively (34). 

A study by Akiyama (2002) on 107-204 pesticide residues in 765 agricultural products of Taiwan showed that 51% of domestic and 32% of imported samples lacked detectable residue and only 2.4% contained more than 5 different residues (8). An investigation of dietary exposure of Thais to pesticides in 8 years (1989-1996) indicated that among 24 pesticides, DDT, dimethoate, methamidophos, and parathion methyl were found annually. However, the dietary intake of all pesticides was far below the established daily intake (17). 

Sannino (2007) reported that in canned peas and tomato and apple juice purees, the highest value (5 mg/kg) was obtained for azadirachtin and avermectin B1b (35). Akiyama *et al*. (2011) from a 15-year monitoring survey (FY 1995-2009) in japan reported that procymidone and iprodione were frequently found in vegetables, and kresoxim-methyl, acetamiprid, iprodione and captan in fruits (8). Likewise, a recent study by Chourasiya *et al.* (2015) reported that the average levels of organochlorine pesticides ranged from 83.8 ± 25.5 ng/g to 222.4 ± 90.0 ng/g for cauliflower from Punjab, India and similarly Szpyrka *et al*. (2015) showed that 36.6% of fruit and vegetables samples from south-eastern Poland were contaminated with pesticides residues (36, 37). 

Furthermore,our findings were agreement with other reports such as Rossato *et al*. (2010), Raina-Fulton *et al. *(2015), and Shamsipur *et al*. (2016) that illustrated the GC-IT-MS method is selective, accurate, precise, and valid for simultaneously evaluating pesticides in fruits and vegetables (38-40).

## Conclusion

The analyzed samples did not contain pesticides residue levels above the accepted maximum residue limits (MRLs) as adapted by the FAO/WHO Codex Alimentarius Commission. Ten pesticides were determined in approximately two-third of the samples. The GC-MS results indicated that the method is an efficient, reliable tool for monitoring pesticide residues in fruits and vegetables. It is important to emphasize that national pesticide monitoring programs that evaluate residues on main crops should be used to routinely estimate the exposure in the Iranian population. This method will likely be widely employed to monitor trace pesticide levels for various fruits and vegetables in future researches.
